# In-vivo oogenesis of oogonial and mesenchymal stem cells seeded in transplanted ovarian extracellular matrix

**DOI:** 10.1186/s13048-023-01131-3

**Published:** 2023-03-20

**Authors:** Leila Mirzaeian, Farideh Eivazkhani, Maryam Saber, Ashraf Moini, Fereshteh Esfandiari, Mojtaba Rezazadeh Valojerdi, Rouhollah Fathi

**Affiliations:** 1 Department of Embryology, Reproductive Biomedicine Research Center, Royan Institute for Reproductive Biomedicine, ACECR, Tehran, 1665659911 Iran; 2 Department of Stem Cells and Developmental Biology, Cell Science Research Center, Royan Institute for Stem Cell Biology and Technology, ACECR, Tehran, Iran; 3 Breast Disease Research Center (BDRC), Tehran University of Medical Sciences, Tehran, Iran; 4 Department of Surgery, Arash Women’s Hospital, Tehran University of Medical Sciences, Tehran, Iran; 5 Department of Endocrinology and Female Infertility at Reproductive Biomedicine Research Center, Royan Institute for Reproductive Biomedicine, ACECR, Tehran, Iran; 6 Department of Anatomy, Faculty of Medical Science, Tarbiat Modares University, Tehran, Iran

**Keywords:** Oogonial Stem Cell, Mesenchymal stem cell, Allograft, Oogenesis, Angiogenesis

## Abstract

**Objective (s):**

One way to overcome the recurrence of cancer cells following ovarian tissue transplantation is to use decellularized tissues as a scaffold that does not have any cellular components. These cell-free scaffolds can be seeded with different type of stem cells for ovarian restoration.

**Materials and methods:**

OSCs, PMSCs and BMSCs (oogonial, peritoneal and bone marrow mesenchymal stem cells, respectively) were seeded into human decellularized ovarian tissue as 4 groups: Scaffold + OSCs (SO), Scaffold + OSC + PMSCs (SOP), Scaffold + OSC + BMSCs (SOB) and Scaffold + OSC + PMSCs + BMSCs (SOPB). The produced grafts were transplanted into the sub-peritoneal space of ovariectomized NMRI mice as artificial ovary (AO). The expression of Vegf, CD34, Gdf9, Zp3, Ddx4, Amh and Lhr genes in AOs were measured by qRT-PCR. Also, histotechniques were considered to detect the anti GFP, PCNA, VEGF, GDF9, ZP3 and AMH proteins.

**Results:**

H & E staining showed follicle-like structures in all groups; the number of these structures, in the SOP and SOB groups, were the highest. In SO group, differentiation ability to oocyte and granulosa cells was observed. Endothelial, oocyte, germ, and granulosa cell-like cells were specially seen in SOP and angiogenesis capability was more in SOB group. However, angiogenesis ability and differentiation to theca cell-like cells were more often in SOPB group. While none of the groups showed a significant difference in AMH level, estradiol levels were significantly higher in SOPB group.

**Conclusion:**

Integration of OSCs + PMSCs and those OSCs + BMSCs were more conducive to oogenesis.

## Introduction

The survival rate of people diagnosed with many types of malignancies is progressively rising, but in many cases, the restoration of fertility in women of reproductive age who have undergone treatment and/or recovery is a serious concern. In women, chemotherapy or radiotherapy treatments can damage the ovaries and cause loss of endocrine and reproductive system functions [1]. Ovarian tissue cryopreservation is currently the only approach to fertility preservation for patients undergoing gonadotoxic treatment [2, 3]. However, this method is not appropriate for patients who have certain types of cancers because there is a risk of the retention of cancer cells in the cryopreserved tissue [4]. A safer method is the encapsulation of isolated follicles in an artificial ovary [5]. This advanced medical technology can be used for women with premature ovarian failure or those who want to delay their fertility or menopause.

In the last decade, decellularization techniques have been applied in regenerative medicine to different tissues and extracellular matrices (ECM) as a platform for tissue or organ remodeling [6]. Lack of cells and reduction of immunogenicity properties in ECM lead to its application as allografts and xenografts. Cell-free ECM not only involves the creation of a three-dimensional skeleton for cell growth but also plays a fundamental role in various biological processes [7]. Ovarian decellularized scaffold can be seeded with stem cells and transplanted after appropriate regeneration through ex vivo culture. Oogonial stem cells (OSCs) and mesenchymal stem cells (MSCs) can be used in this way to obtain oocytes and follicles.

In recent years, there is evidence of oogenesis and the presence of Oogonial Stem Cells (OSCs) can lead to some extent of regeneration in mature mammalian ovaries [8]. Hence, these cells can potentially be used to prevent decrease in follicle numbers with aging and menopause. Cell lineage analysis of OSCs in the rodent ovary suggests that oogenesis probably continues postnatally [9]. A study of ovarian follicle numbers in the adult murine shows that postnatal follicular reconstruction is necessary to maintain primordial follicle reserves, indicating that stem cells may exist in the mammalian ovary [10]. Virant-Klun et al. showed the presence of pluripotent stem cells in ovarian surface epithelium in postmenopausal women and those with Premature Ovarian Failure (POF) [11]. Some POF models posit that fertility is reversible, confirming that there are probably oogonial stem cells in the ovary.

In recent years, Mesenchymal Stem Cells (MSC) have been considered for treatment of various diseases including infertility [12]. MSCs are adult stem cells that do not yet function and are isolated from many tissues. MSCs have been used in the treatment of many diseases in recent years due to their high proliferation ability, simple isolation, low immunogenicity, and absence of ethical constraints. MSCs differentiate into specific tissues and organs under certain conditions with great differentiation potential into many types of cells [5]. Studies have shown that MSCs are capable of differentiation into germ cell-like cells after transplantation to gonadal tissue. Zhang et al. observed the improvement of serum hormonal level, follicular development, reduction of follicular atresia and granulosa cells apoptosis and recovery of ovarian reserve capacity after transplantation of MSCs isolated from human placenta into adult POF mice. It is believed that the production and secretion of cytokines by MSC regulates Anti-Mullerian Hormone (AMH) expression in granulosa cells, and prevents the apoptosis of granulosa cells [13].

The differentiation of oogonial stem cells (OSCs) into oocytes, on the other hand, requires interaction with somatic cells. Ovarian somatic cells play a major role in germ cells development in the ovaries and may be effective in the viability and differentiation of OSCs [14]. MSCs along with OSCs, as somatic cells, or by themselves, could potentially differentiate into ovarian cell-like cells. This study is aimed at understanding oogenesis from MSCs.

In this work, Peritoneum Mesenchymal Stem Cells (PMSCs) and Bone Marrow Mesenchymal Stem Cells (BMSCs), along with OSCs, were seeded into human decellularized ovarian scaffold, and the construct transplanted to sub-peritoneum ovariectomized immunodeficient and non-immunodeficient mice for oogenesis induction.

## Materials and methods

### Preparing, labeling, and seeding the cells

The present study was performed in compliance to the rules of the research ethical committee of Royan Institute (IR.ACECR.ROYAN.REC.1398.54). The experimental groups were human decellularized ovarian scaffold seeded with three types of mouse cells (OSCs, PMSCs, and BMSCs).

### Preparing the GFP-positive OSCs

Green Fluorescent Protein (GFP)-positive OSCs were isolated from ovarian tissues of transgenic mice (6–8 weeks old, Royan Institute, Tehran, Iran) expressing GFP with chicken beta-actin promoter and cytomegalovirus enhancer (pCAG). For every isolation, ovaries from 4 transgenic mice were collected and OSCs were isolated by the enzymatic digestion method. Dissected ovaries were placed in the enzymatic solution containing the collagenase type IV (800 U/ml, prepared in HBSS) and DNase-I (1 μg/ml, Sigma-Aldrich) and gently agitated in a water bath (37 °C) for 15 min. Then, the supernatant was collected into a new 15-ml conical tube and this incubation continued until no visible pieces of ovary were present. The collected supernatants were filtered through a 40-μm nylon mesh and centrifuged at 300 g for 5 min and washed twice. The supernatant was removed, and the cells were re-suspended in culture medium. Isolated cells were plated onto gelatin-coated culture plates (3 × 105 cells/per 3 cm2 plate, Falcon) with the culture medium containing α-MEM (Invitrogen) supplemented with 10% FBS (Hyclone), 1 mM sodium pyruvate (Invitrogen), 1 mM nonessential amino acids (Invitrogen), 1X penicillin–streptomycin-glutamine (Invitrogen), 0.1 mM β-mercaptoethanol (Sigma-Aldrich), 1X concentrated N-2 supplement (R&D), 103 units/ml leukemia inhibitory factor (LIF, Royan Institute), 10 ng/ml recombinant human epidermal growth factor (rhEGF, Royan BioTech), 1 ng/ml basic fibroblast growth factor (bFGF, Royan BioTech), and 40 ng/ml glial cell-derived neurotropic factor (GDNF, Royan BioTech). After 30 min to 1 h of culture at 37 °C, the supernatant that contained cells not attached to the plate were collected and transferred to a new 24-well plate coated with feeder cells (mitomycin C-treated MEFs). The medium was changed every 2–3 days and when the cells reached confluence on the plate, they were digested with 0.05% trypsin (Invitrogen) followed by neutralization by adding 10% fetal bovine serum (FBS) and replated on fresh MEF at a 1:2 split ratio. Identification and characterization of OSCs were performed in a previous work [15].

### PKH labeling of reseeded cells

PKH26 Red is a fluorescent molecule that attaches to cell membrane. 5 × 10^6^ of the single-cell PMSCs or BMSCs were labeled 1 h prior to their application. The method of PMSCs and BMSCs extraction was based on previous works [16, 17]. They were rinsed with serum-free DMEM/F12. After centrifuging (1600 rpm, 20–25˚C, 5 min), 250 μl diluent C was added to the cell plate and re-suspended with gentle pipetting. 1 μl of PKH26 mixed with 250 μl diluent C were added to the solution and pipetted carefully for uniform staining and reproducible cell labeling for 5–10 min. Then, 1 ml of fetal bovine serum (FBS) was added to stop staining. The cells were centrifuged at 1600 rpm, 20–25˚C for 5 min. To ensure removal of unbound dye, the cell plate was washed with serum free DMEM/F12 and cell centrifugation was carried out at 1600 rpm, 20–25˚C for 5 min. Then, DMEM/F12 (Gibco) was enriched with 15% fetal bovine serum (FBS) (Gibco), 1% non-essential amino acids 100 × (Gibco), 1% Glutamax (Gibco), 1% insulin transferrin selenium (ITS) (Gibco) and 1% (5000 units/ml) penicillin/streptomycin (Thermo) and added to cell plate, pipetted, and stained cells observed with fluorescence microscopy.

### Labeling of reseeded cells with SPIO and Prussian blue staining

Once BMSCs reached 70–80% confluency and one hour before use, the cells were exposed and labeled with 100 µg/ml superparamagnetic iron oxide nanoparticles (SPIO) supplemented with 40 μg/mL concentration of protamine sulphate for 24–48 h in vitro. The BMSCs were rinsed twice with PBS. SPIO is a non-fluorescent particle that enters the cell through phagocytic absorption.

### Prussian blue staining technique used for confirming the presence of SPIO nanoparticles within the cells

BMSCs that adhered to the bottom of the tissue culture after staining with SPIO, were washed with phosphate buffered saline (PBS) twice to remove free iron particles. They were fixed with mixing solution containing hydrochlorc acid and potassium ferrocyanide (1:1) for 10 min. After being washed with distilled water, the cells were exposed to 2% pararosaniline dye for SPIO staining for 5 min and were rinsed with distilled water. The cells can be observed with membranes or dark blue cytoplasm by an invert microscope.

### Prussian blue staining for tissues

Prussian blue staining technique was used for detecting the SPIO nanoparticles and determining their location within the artificial ovaries after the duration of culture. 6 µm tissue sections were deparaffinized and hydrated by xylene. A 1:1 mixture of hydrochloric acid and potassium ferrocyanide was used as fixative solution for 10 min. After rinsing with distilled water, tissue staining was performed using 2% pararosaniline dye treatment for 5 min. The tissues were washed, dehydrated with increasing concentration of ethanol (70–100) and cleared with xylene. After mounting, the tissue sections were observed using an upright microscope.

### Cellular seeding into human ovarian scaffold and group design

Human ovarian samples were obtained from 18–22 years old transsexuals (Arash Hospital,Tehran, Iran). Decellularization of ovary and production of ovarian scaffold was performed as mentioned in our previous work [18]. For each group, 12 human ovarian scaffolds (5 × 5 × 1 mm) were used as follows:

In group 1 (SO; Scaffold + OSCs) OSCs were seeded into human ovarian scaffolds. OSCs (GFP^+^) were passaged and after cell counting with a Neubauer Chamber, 62.5 × 10^3^ cells in DMEM/F12 (Gibco) enriched with 15% fetal bovine serum (FBS) (Gibco), 1% non-essential amino acids 100 × (Gibco),1% Glutamax (Gibco), 1% insulin transferrin selenium (ITS) (Gibco), 1% (5000 units/ml) penicillin /streptomycin (Thermo) were injected using insulin syringe inside each scaffold. Thus, in this group, 12 scaffolds with one cell type (75 × 10^4^) were placed in a spinner flask and stored with stirring at 20 rpm for one week under incubator conditions. In group 2 (SOP; Scaffold + OSCs + PMSCs) and group 3 (SOB; Scaffold + OSCs + PMSCs), PMSCs (PKH^+^) and BMSCs (SPIO^+^) were used along with OSCs in a ratio of 10 to 1 respectively. But in group 4 (SOPB; scaffold + OSCs + PMSCs + BMSCs), all 3 types of cells (PMSCs, BMSCs and OSCs) in a 5:5:1 were seeded into each scaffold.

### Allotransplantation of ovarian recellularized scaffold

**Fig. 1:**
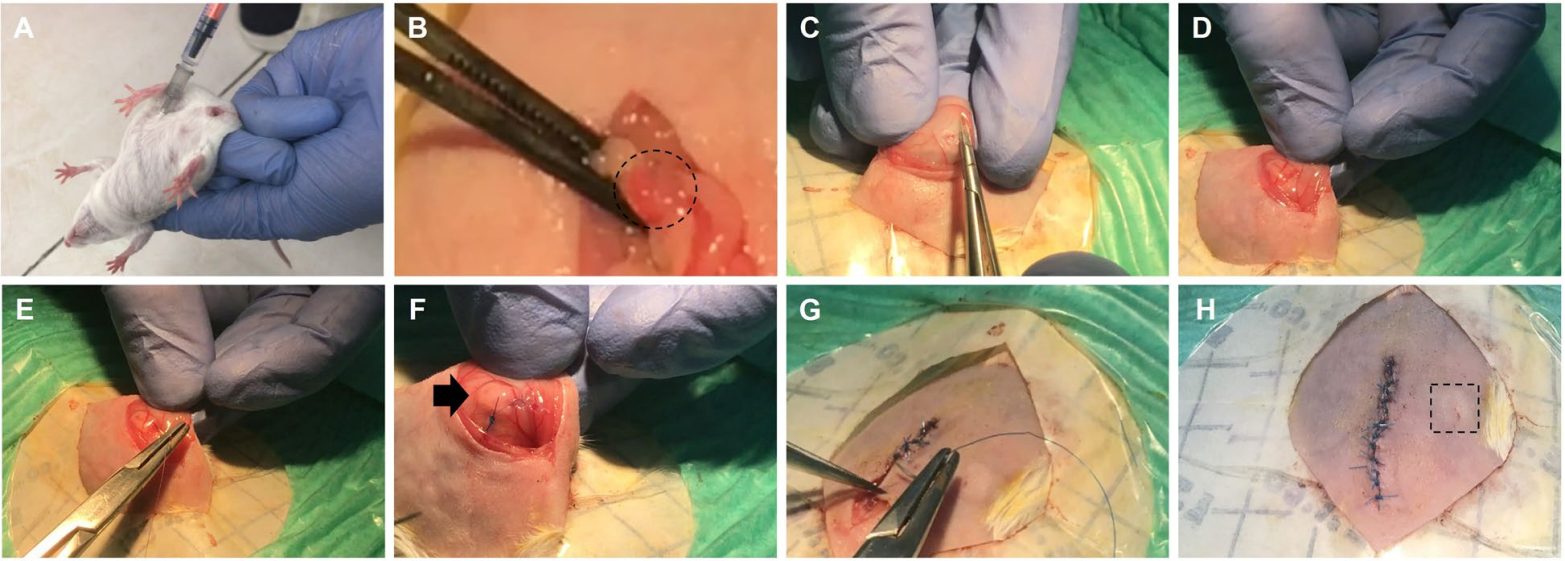
Ovarian engineered constructs transplantation. Ovarian engineered constructs were transplanted into adult female mice (NMRI). **A** Mice were first anesthetized via intra-peritoneal injection of mixture of a 100 mg/kg of ketamine and 15 mg/kg of xylazine. **B** The ovaries (dashed circle) were excited, and (**C**-**F**) the constructs placed within a small pocket created within anterior abdominal wall peritoneum mesothelium and then sutured (6–0 Nylon) in place. **G** Gross histology of grafts with cells under anterior abdominal wall peritoneum mesothelium (Arrow) and (**H**) under the skin (dashed square)

Ketamine/xylazine was injected with an insulin syringe into the peritoneum for anesthesia of adult (6–8 weeks) NMRI mice (Fig. 1A). Iodine scrub was spread over the surface of the abdominal skin, which was then shaved. Then, a fissure was made over the abdominal skin and subcutaneous layers. Abdominal lipid attached to the ovaries were removed from the body cavity and the ovaries were inserted using the top of a uterine horn (Fig. 1B). Following ovariectomized surgery, a small incision was made on the inner side the anterior abdominal wall peritoneum mesothelium to create a pocket. The recellularized scaffolds were gently slid into the peritoneum mesothelium pocket and incision edges were closed with absorbable sutures 6/0. To determine the location of the graft region, 1 staple was sutured with a 6/0 non-absorbable suture near the scaffold (Fig. 1C-F). Then subcutaneous layers were continuously closed with absorbable sutures 6/0. The skin with non-absorbable suture 6/0 was overlapped (Fig. 1G and H). The transplanted mice were maintained under animal conditions for 8 weeks. Control animals with natural cycle that were not subjected to surgery and ovariectomized mice that did not undergo transplantation were used for vaginal opening and serum hormone studies. All of these experiments were conducted in triplicates. The transplantation into immunosuppressed mice was performed for the SOP group. All artificial ovaries were examined for histology with H & E staining at 2, 4, 6 and 8 weeks after transplantation.

### Hormonal assays

Estradiol and AMH were measured in blood serum on mice that were ovariectomized and received ovarian recellularized with stem cells for 4 groups at 8th week using commercial ELISA Kits (Bioassay Technology Company of China) [19].

### RT-qPCR analysis

The mice with transplants were anesthetized after 8 weeks and blood sampling was taken from the heart. Left artificial ovary was stored in RNA latter at -196° C. Freezed artificial ovaries were entirely dried from RNA latter, chopped and transferred into 1.5 mL microtubes with TRIzol reagent according to the manufacturer’s protocol. Briefly, for RNA extraction from tissues, the sample was homogenized with TRIzol reagent (500 µL/artificial ovary) for 20 min. To separate RNA from TRIzol, chloroform (100µL) was added to homogenized solution and shaken for 10 min and centrifuged (1200 rcf, 4˚C, 15 min). After clear top phase containing RNA was transported to new mircotube, isopropanol was added to settle the RNA. They were then incubated overnight at 20˚C. The solution with RNA was centrifuged before (1200 rcf, 4˚C, 15 min) and after (7500 rcf, 4˚C, 8 min) addition of 1 mL 70% ethanol followed by pipetting the RNA pellet in DEPC water. Total RNA concentration was measured using spectrometry at 260 nm. Then cDNA Synthesis Kit (TaKaRa) was used for cDNA synthesis and subsequent RT-qPCR was performed in duplicate on each sample for determination and comparison of transcript level of eight genes listed in Table 1. Finally, using comparative threshold cycle number (2^−ΔCt^) method, gene expression level was determined in each artificial ovary.

**Table 1:**
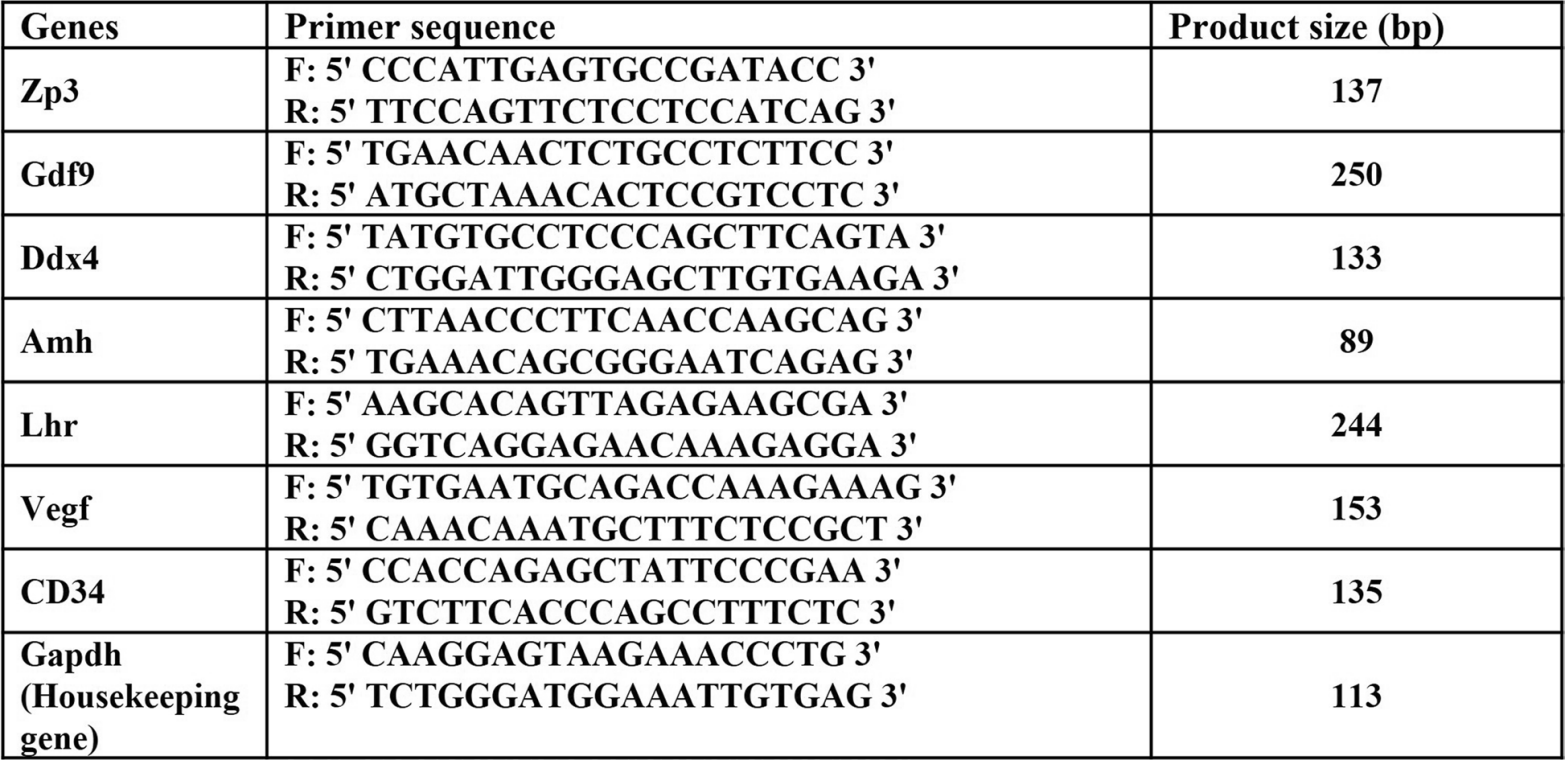
Primers used in Qrt-PCR

### Simple histology (H&E) staining

Right artificial ovary was fixed in Bouin's (24 h), and transferred to 10% formalin buffer (1–7 days) for histological examination. The samples were shaken daily. Artificial ovaries were isolated from the body and after processing, serially cut into 6 μm thickness. Every sixth tissue section was stained with hematoxylin and eosin to detect structures formed by cellular differentiation indicating oogenesis and angiogenesis in ovarian scaffolds after transplantation. Other sections were preserved for immunohistochemical staining.

### Immunohistology assay

Immunohistochemistry staining was performed for evaluation of oogenesis and angiogenesis markers and proteins development in artificial ovary after transplantation. For this purpose, 6 μm sections were serially mounted on a charged chamber, and placed for 30–40 min at 60˚C. After deparaffinization in xylene and hydration in descending concentration levels of ethanol (100–70), buffered sodium citrate (PH: 6, 90˚C for 60 min) was used for antigen retrieval. After cooling the slides to room temperature, sections were rinsed with PBS-tween (0.05%). For peroxidase-linked immunostaining, endogenous peroxidase was deactivated with 10% hydrogen peroxide (H2O2) for 30 min followed by three-time retrieval and double washing in PBS-tween. For penetrability in membrane, tissue sections were treated with Triton X-100 (0.5%) for 15 min. Non-specific antigens were blocked with 10% secondary host serum at 37˚C for one hour followed by soaking twice in PBS-tween. Slides were incubated with primary antibodies including anti-GDF9 (1:100, RAB0104, Santa Cruz), anti-ZP3 (1:100, RAB05146, abcam), anti-AMH (1:100, GTX129593, cell signaling) anti- PCNA (1:100, BD610665, BD) and anti-GFP (1:100, 338,002, Biolegend) at 4˚C, overnight. Then, the slides were immersed thrice in PBS-Tween, and incubated for one hour with goat anti-rabbit (1:500, 65–6120, Invitrogen), rabbit anti-goat (1:500, ab6741, abcam), rabbit anti-rat (1:500, a5795, sigma) and Goat Anti-mouse (1:500, ab6789, abcam) IgG HRP labeled secondary antibodies and washed thrice in PBS-Tween for 45 min with three-times exchange followed by treatment with substrate chromogen solution (DAB) reagent (ABC; detection IHC kit) at room temperature in the dark for 5–20 min. Then, hematoxylin staining was performed for cellular counterstaining. In the negative control, the sections were incubated in PBS instead of primary antibody and prepared to rule out nonspecific labeling. After mounting under coverslips, the sections were evaluated using light microscopy (Olympus IX51, Japan).

### Statistical analysis

The statistical differences between the groups were analyzed using IBM SPSS Statistics for Windows, Version 22.0 (IBM Crop., Armonk, NY, USA). In the present study, continuous variables were expressed as Mean ± SEM. One-way ANOVA (Duncan's complementary test) or Kruskal–Wallis (Dunn's complementary test) tests were used to assess differences between the groups. Two-sided statistical tests with *P* < 0.05 was considered statistically significant.

## Results

### Microscopic observation of stem cells morphology before seeding

**Fig. 2:**
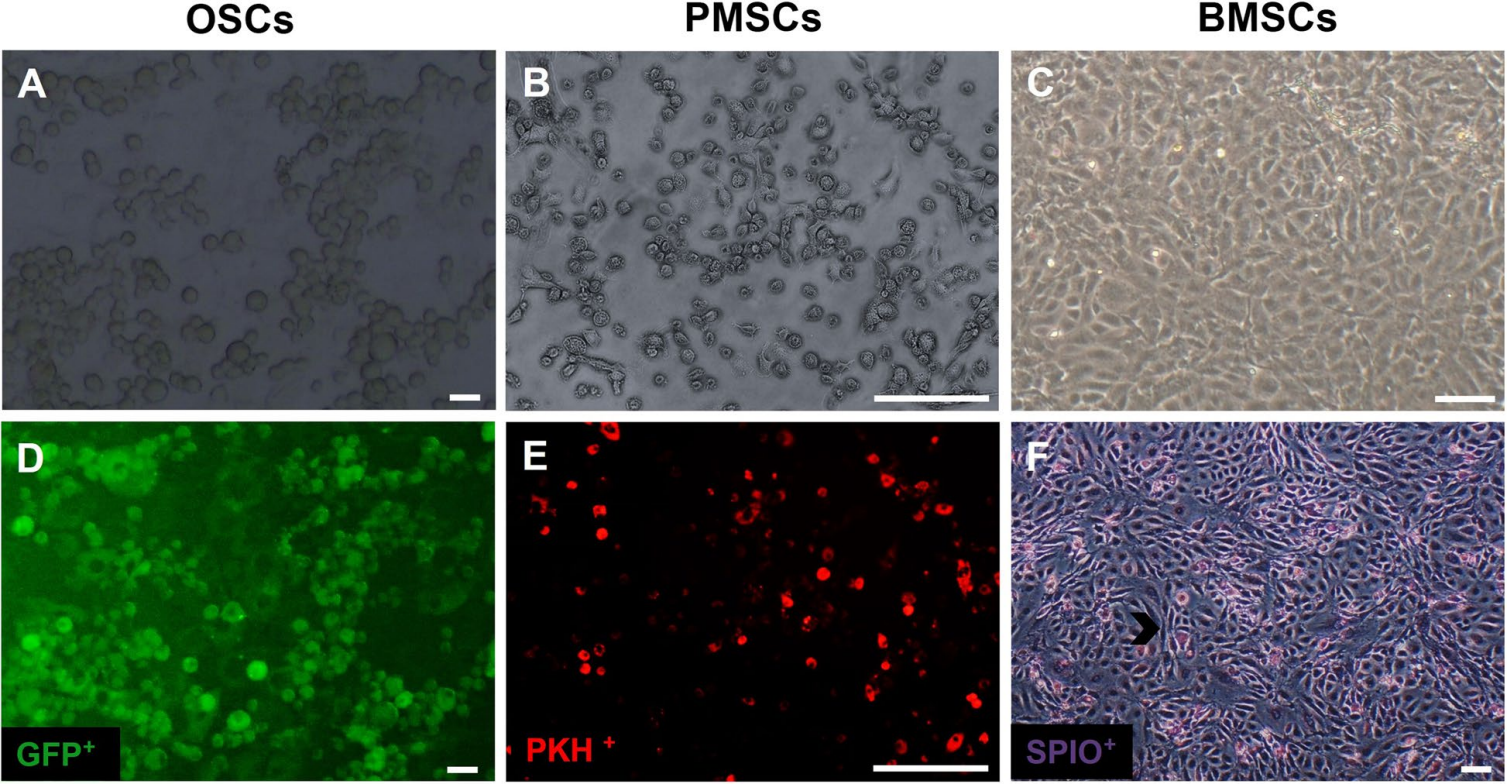
Microscopic observation of stem cells pre-cellular seeding. OSCs (**A** and **D**) and PMSCs (**B** and **E**) or BMSCs (**C** and **F**) culture before seeded into decellularized ovarian scaffold. Distribution of GFP^+^ OSCs (green) and PMSCs (red) which observed by fluorescent microscopy (**D** and **E**) and BMSCs (purple-blue) after stained with Prussian blue that shown by light microscopy (F; SPIO labeled: chevron) (scale bars: 100 µm)

Non adherent culture, spherical shape morphology and green color of OSCs (GFP^+^) were observed under light and fluorescence microscopy (Fig. 2A and D). Spindle morphology similar to MSCs and adherent culture of PMSCs were seen under light microscopy (Fig. 2B). These cells were labeled with PKH red marker and detected using fluorescent microscopy (Fig. 2E).

BMSCs have been extensively used in cell therapy in recent years. These cells play a role in differentiating into tissue-specific cells and angiogenesis induction. Figure 2C shows the characteristics of plastic adherence morphology in BMSCs as seen in light microscopy. These cells were labeled with SPIO and seen in purple using the Prussian blue staining technique (Fig. 2F).

### Microscopic observation of stem cells morphology after seeding

**Fig. 3:**
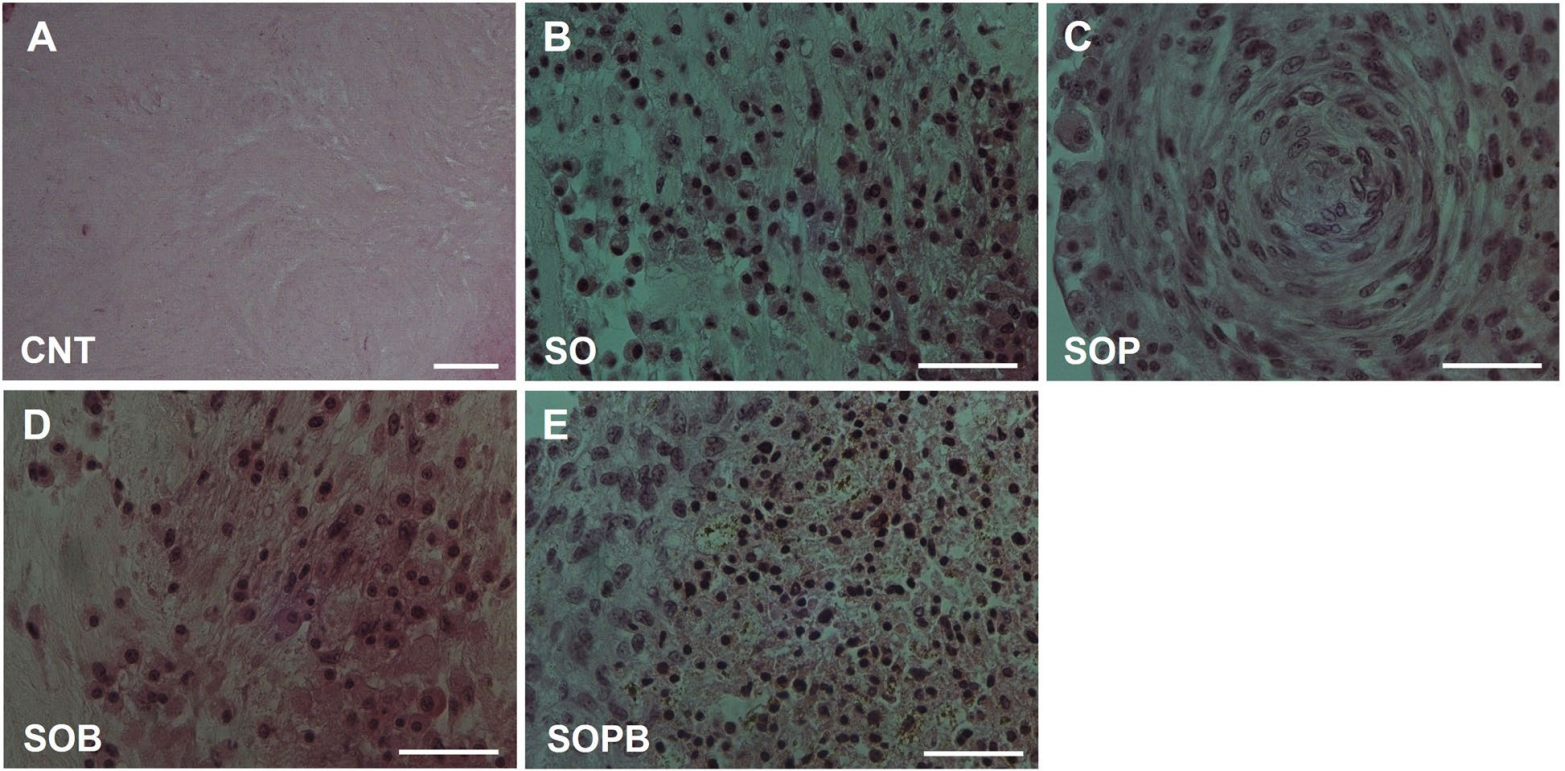
H & E staining of recellularized human ovarian matrix with OSCs; Scaffold without cells as the control group (**A**), SO group (**B**), OSCs + PMSCs; SOP group (**C**), OSCs + BMSCs; SOB group (**D**), OSCs + PMSCs + BMSCs; SOPB group (**E**) after seeding (Scale bars: 50 µm)

**Fig. 4:**
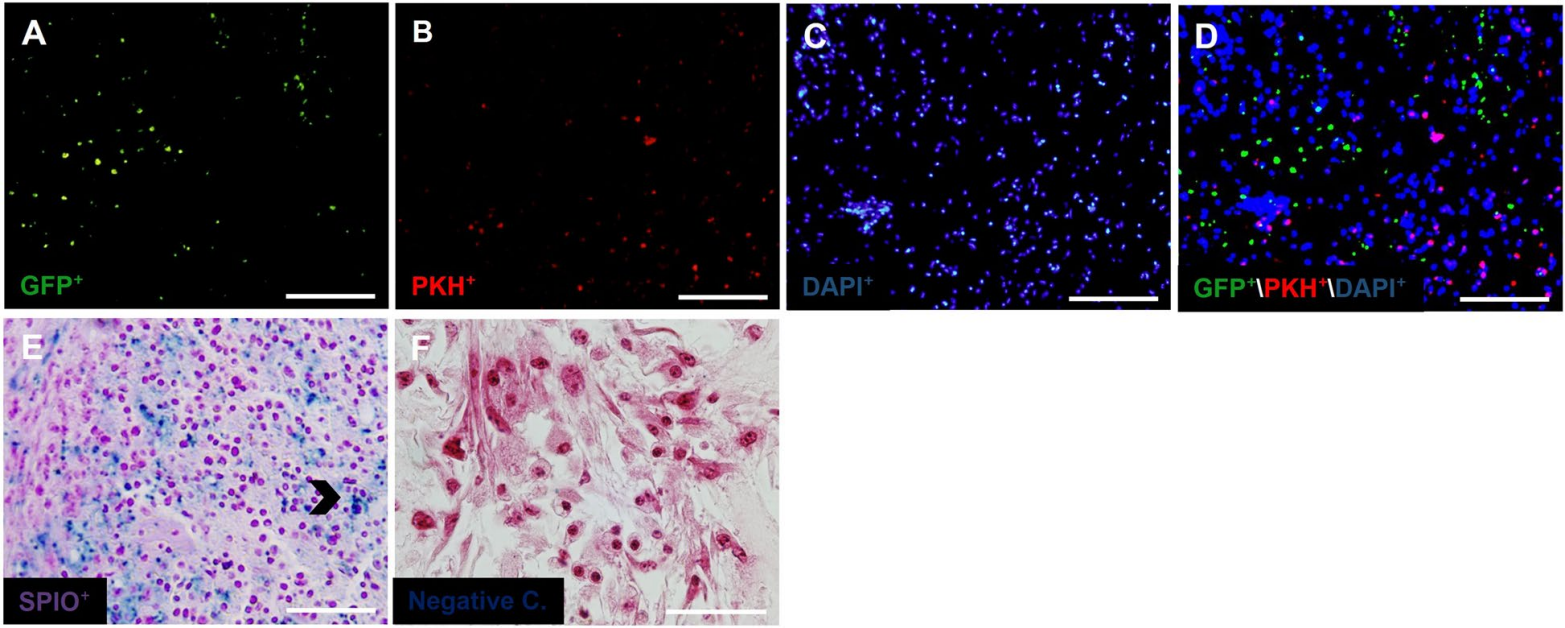
Images of stem cells seeded into decellularized ovarian scaffold. Distribution of GFP^+^ OSCs (green) and PKH^+^ PMSCs with fluorescent microscopy (**A**-**D**). Prussian blue staining (**E**; chevron) and for BMSCs labeled with SPIO and negative control (**F**). (scale bar: 50 µm)

The scaffold without cells and morphology of seeded cells into the ovarian scaffolds could be seen after H & E staining (Fig. 3A-E). As seen in the figure, these cells form spherical assemblages in the ovarian matrix. Since each of the seeded cells in the ovarian scaffold is labeled with a specific marker, OSCs (green) and PMSCs (red) were observed after seeding using fluorescence microscopy (Fig. 4A-D). It was also necessary to show that after seeding, in addition to the red and green cells, BMSCs labeled with SPIO penetrated into scaffolds. Prussian blue staining showed the presence of these cells within the scaffolds. Negative control group indicates the specificity of the Prussian blue staining (Fig. 4E and F). Hence, all three types of stem cells used for seeding were able to penetrate into the ovarian scaffold.

### Puberty resumption in mice after transplantation of artificial ovary

**Fig. 5:**
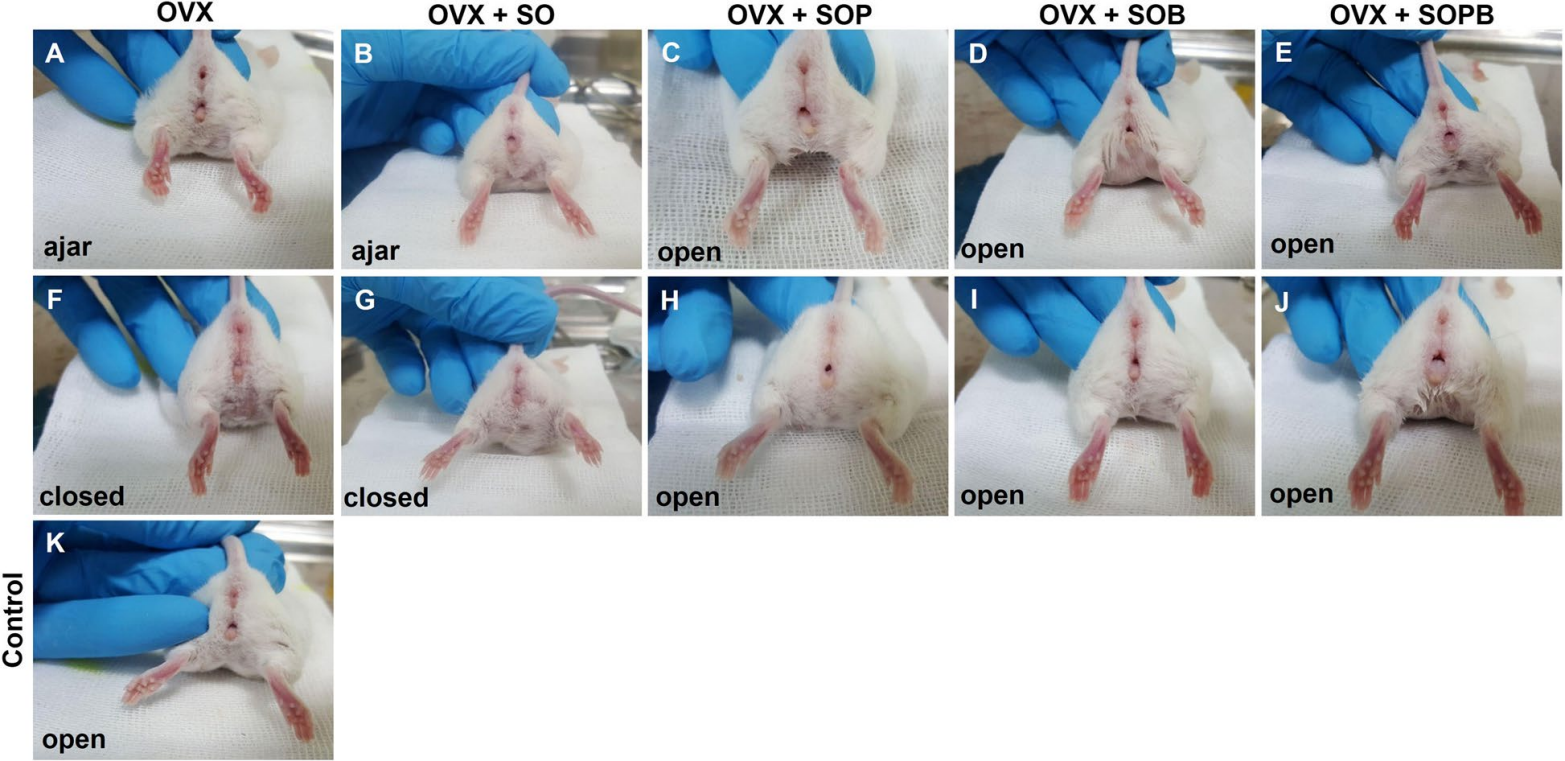
Vaginal opening as an indicator of oogenesis induction. Mice were ovariectomized (OVX, **A**-**J**). Human ovarian scaffolds with OSCs; SO (**B** and **G**) OSCs + PMSCs; SOP (**C** and **H**), OSCs + BMSCs; SOB (**D** and **I**), OSCs + PMSCs + BMSCs; SOPB (**E** and **J**) were surgically placed sub-peritoneum. Mice were analyzed 8 weeks later and the vaginal opening was compared to the control animal (**K**). The mice with the seeded graft including of OSCs together MSCs had open vaginals like the control (**K**), while the OVX mice did not (**A** and **F**)

Mice were ovariectomized to determine whether ovarian decellularized scaffolds could retain endocrine function in vivo. The scaffold was seeded with stem cells and as previously mentioned, the ovarian construct was transplanted to mouse sub-peritoneum region (subserosal fascia) for 8 weeks. The primary endpoints were the serum hormone levels and vaginal opening. Based on mouse maturity criteria, SO group were similar to those of the ovariectomized mice. But in the SOP, SOB and SOPB groups, vaginal orifices usually opened (Fig. 5).

**Fig. 6:**
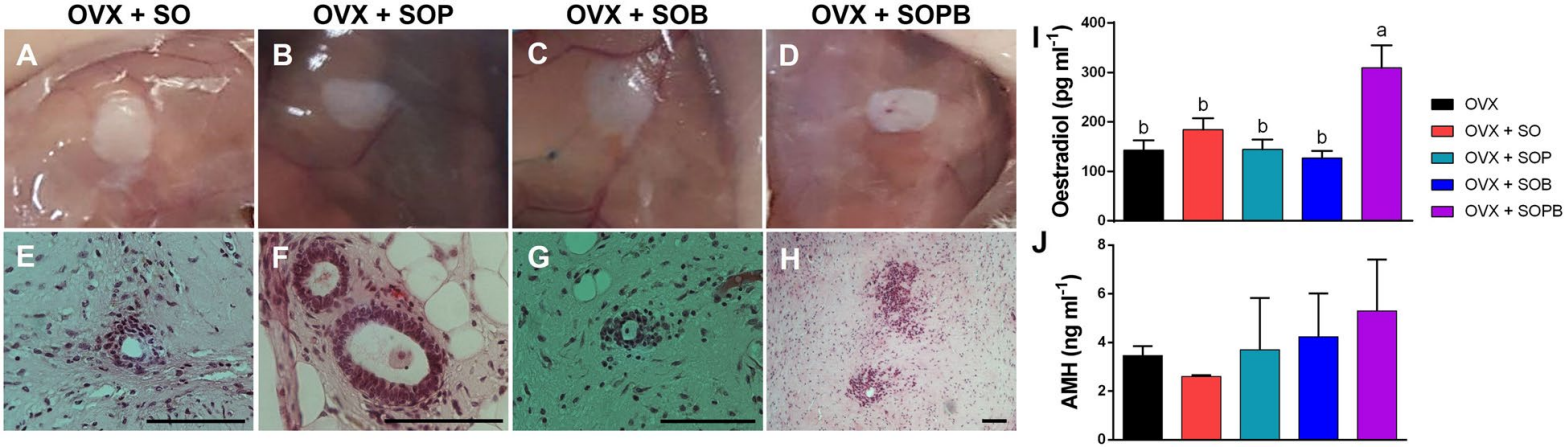
Stem cells cultured on decellularized ovarian scaffold transplanted to mice and produce estradiol and Amh in vivo. **A**-**D** Macroscopic appearance of artificial ovaries with OSCs; SO (**A**), OSCs + PMSCs; SOP (**B**), OSCs + BMSCs; SOB (**C**), OSCs + PMSCs + BMSCs; SOPB (**D**) 8 weeks after transplantation under sub-peritoneum. (**E**–**H**) H&E staining of sections through graft containing follicles-like structures derived from artificial ovaries with OSCs (**E**), OSCs + PMSCs (**F**), OSCs + BMSCs (**G**) and OSCs + PMSCs + BMSCs (**H**) 8 weeks after transplantation. (**I** and **J**) The levels of estradiol and AMH were determined by ELISA in all groups (scale bars: 100 µm). Values are given as mean ± SE. (a-b) Groups followed by the same letter are not significantly different at *p* < 0.05

It is important to note that these grafts were transplanted to NMRI non-immunodeficient mice. Thus, to assess the general health of grafts, their gross histology should be examined at the site of the transplantation (Fig. 6A-D). It seems that graft rejections were not observed in the peritoneum and the fibrous capsule was not formed. As seen in Fig. 11 angiogenesis around the grafts, especially in SOP and SOB groups are clear, and it can be understood that blood supply has been provided in the mentioned groups. All grafts were serially sectioned and stained with H & E staining technique. Follicle-like structures were observed in all four groups of transplanted mice. This condition was more evident in SOP group (Fig. 6E-H).

Serum estradiol levels were measured in ovariectomized and grafted mice and SOPB showed a significant increase compared to the other groups. (Fig. 6I). Furthermore, anti mullerian hormone was detected in all groups, but no significant differences were observed (Fig. 6J).

### Microscopic observation of transplanted labeled stem cells

**Fig. 7:**
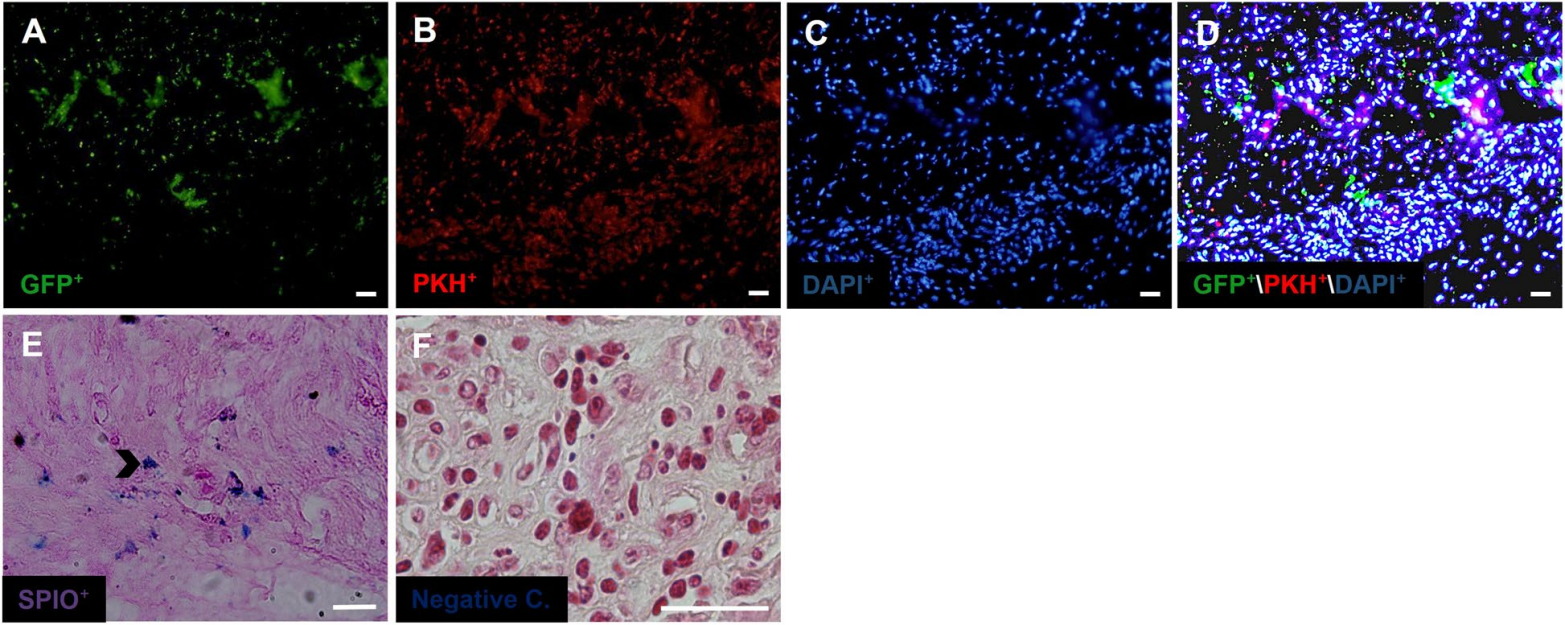
Images of position of labeled stem cells in artificial ovary after transplantation using light and fluorescent microscopy. Distribution of GFP^+^ OSCs (green) and PKH^+^ PMSCs with fluorescent microscopy in (**A**-**D**). Prussian blue positive BMSCs labeled with SPIO (**E**; chevron) and negative control (**F**: Negative C), (scale bar: 20 µm)

Prior to and post cellular seeding into the scaffold, microscopic examination of the cells ensured that all three types of seeding cells penetrated the ovarian scaffold. On the other hand, after transplantation, it is also necessary to show that the labeled cells are present within the artificial ovary. Each type of stem cells was labeled with a specific marker, and after completion of transplantation duration, OSCs (GFP^+^) and PMSCs (PKH^+^) were observed using fluorescence microscopy (Fig. 7A-D). BMSCs)SPIO^+^) were stained with Prussian blue in addition to OSCs and PMSCs existed in the artificial ovary and were observed by light microscopy. The presence of a negative control indicates the specificity of Prussian blue staining (Fig. 7E and F). Thus, all three groups of stem cells have been able to survive during xenotransplantation in artificial ovary.

### Evaluation and comparison of the expression of specific genes in endothelial, oocyte, germ, granulosa, and theca cells

**Fig. 8:**
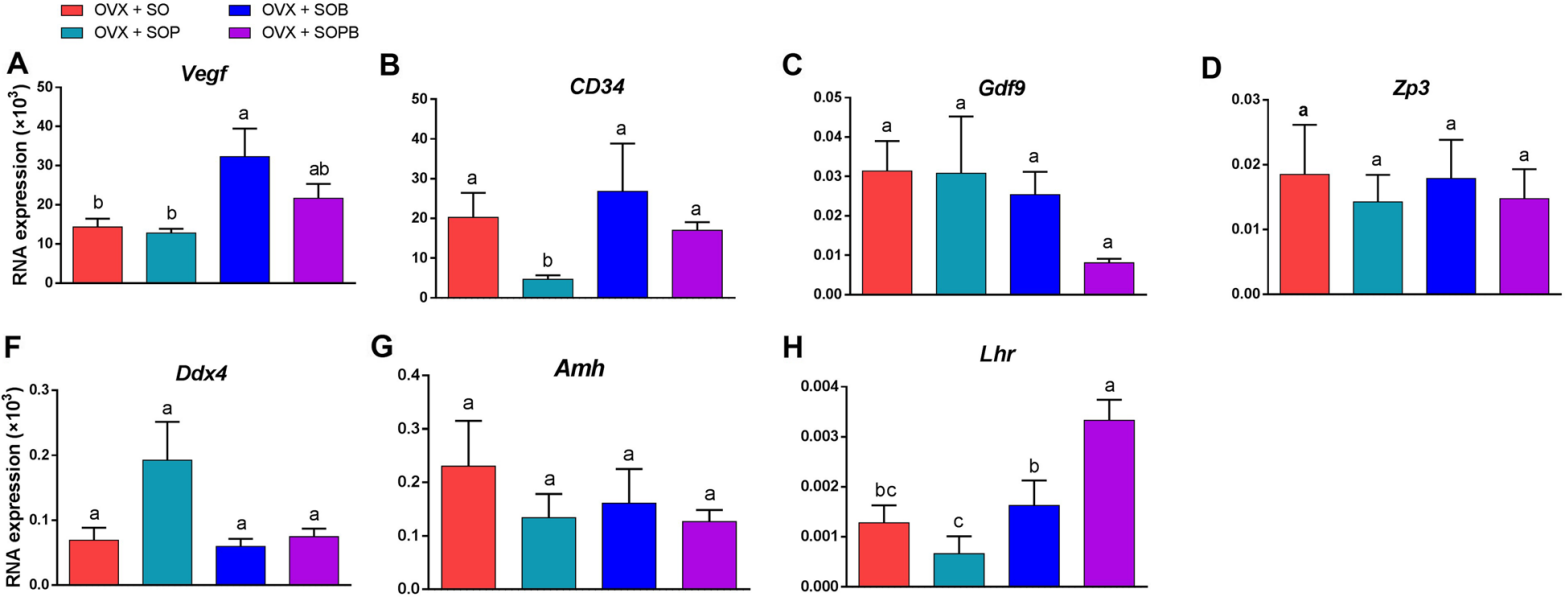
Analysis of gene expression in endothelial cells for angiogenesis (Vegf, CD34), oocyte cell (Zp3, Gdf9), germ cell (Ddx4), granulosa cell (Amh) and theca cell (Lhr) markers in Artificial ovary by RT–qPCR. Gene expression in differentiated cells determined using the comparative threshold cycle number (2^–ΔCt^) method. Values are given as mean ± SEM. (a-b) Groups followed by the same letter are not significantly different at *p* < 0.05

At the level of angiogenesis, SOB group showed significant rise in Vegf gene expression, compared to SO and SOP groups and about CD34 marker, SO, SOB and SOPB presented significant elevation compared to SOP group (*P* < 0.05). The expressions of genes in oocyte and granulosa cells did not show significant differences between the groups. Expression of Ddx4 gene increased more in the SOP group compared to the other groups, but not to a significant extent. However, Lhr expression showed statistical rise in SOPB group compared to the other groups (Fig. 8).

### Immunohistochemical staining to detect and localize GFP, PCNA, VEGF, GDF9, ZP3, DDX4 and AMH proteins

**Fig. 9:**
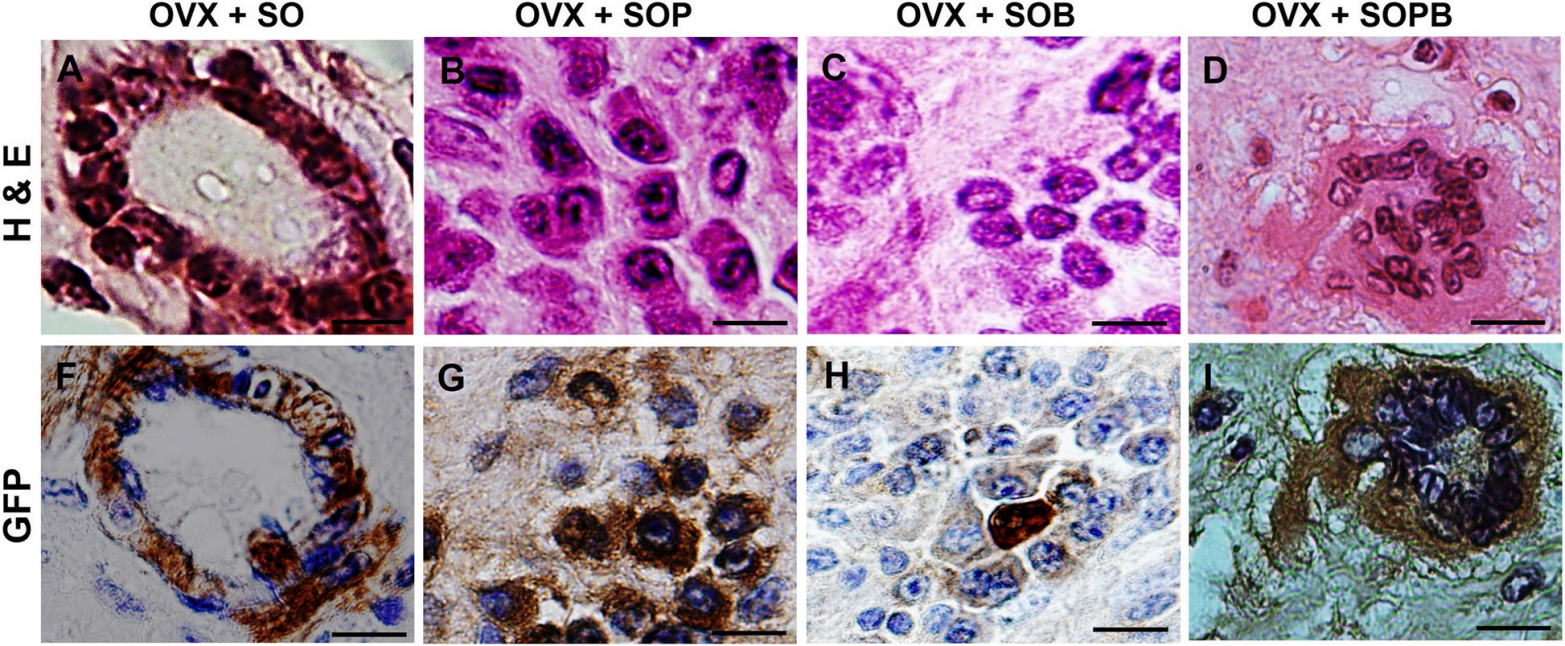
H & E and Immunohistochemical staining for antibodies against Green fluorescent protein (GFP*)* protein in artificial ovaries (Scale bars: 10 µm)

**Fig. 10:**
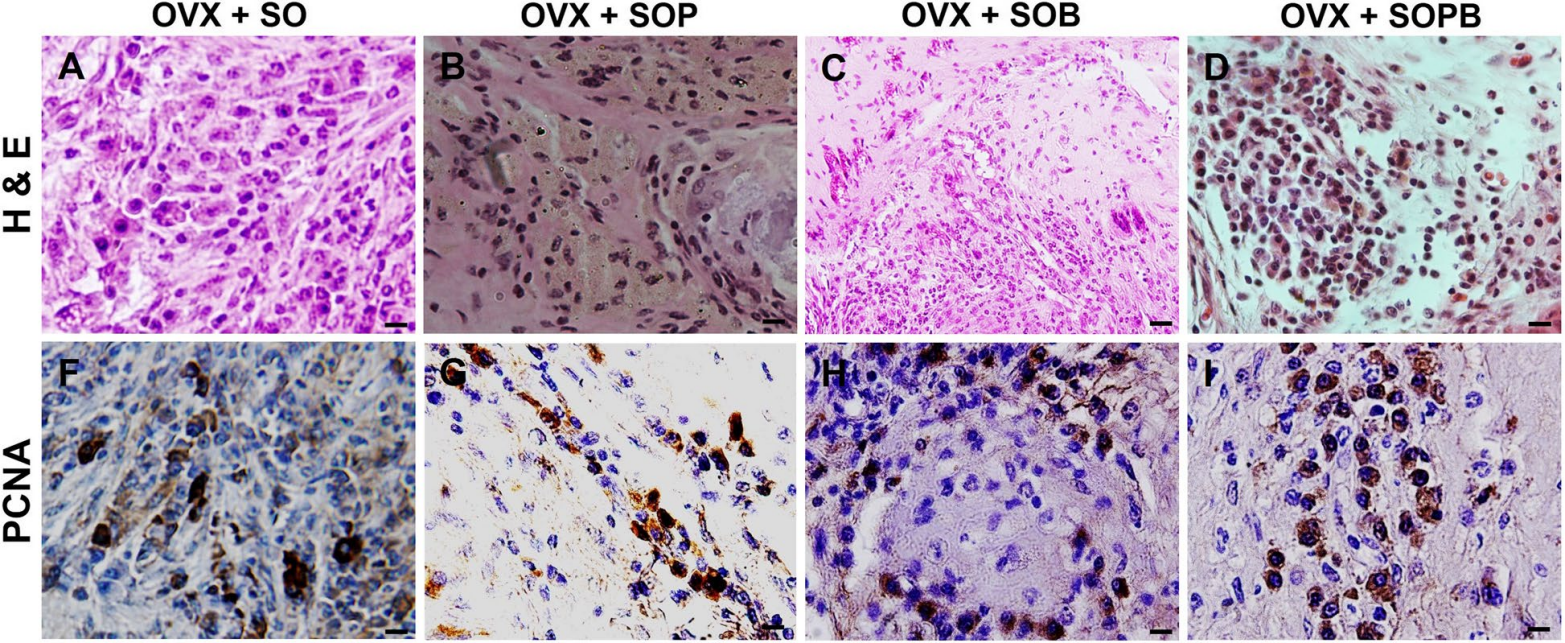
H & E and Immunohistochemical staining for antibodies against Proliferating cell nuclear antigen (PCNA) protein in artificial ovaries (Scale bars: 10 µm)

**Fig. 11:**
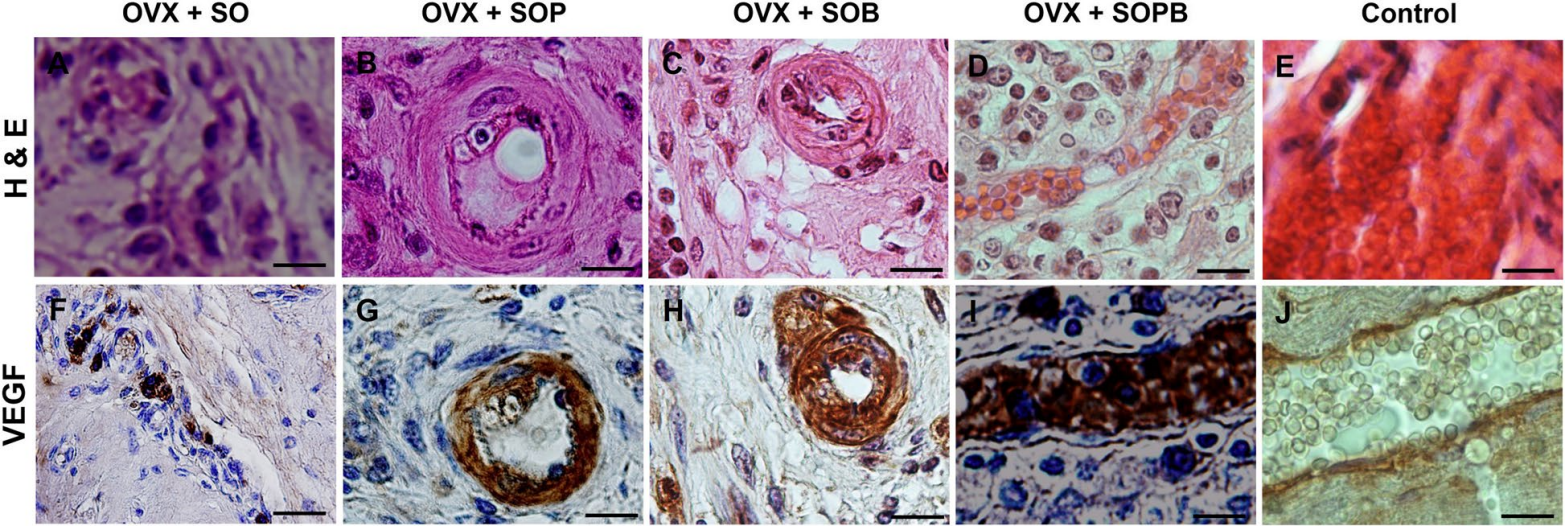
H & E and Immunohistochemical staining for antibodies against Vascular endothelial growth factor (VEGF*)* protein in artificial ovaries (Scale bars: 10 µm)

**Fig. 12:**
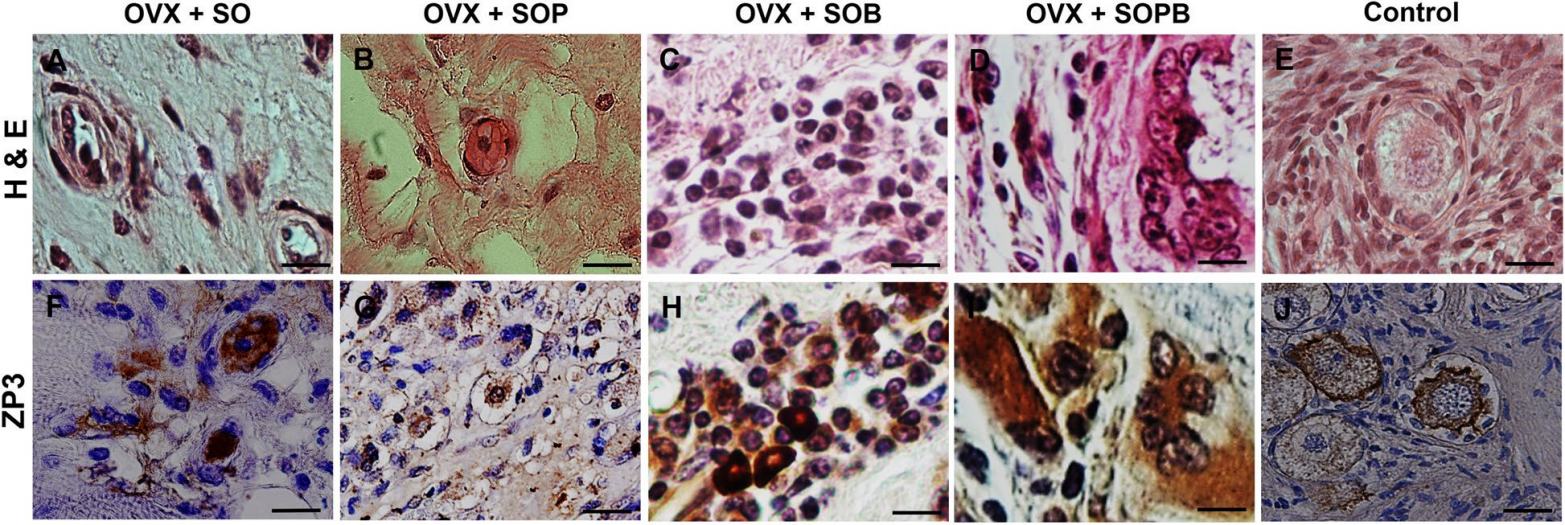
H & E and Immunohistochemical staining for antibodies against Zona pellucida glycoprotein 3 (ZP3*)* protein in artificial ovaries (Scale bars: 20 µm)

**Fig. 13:**
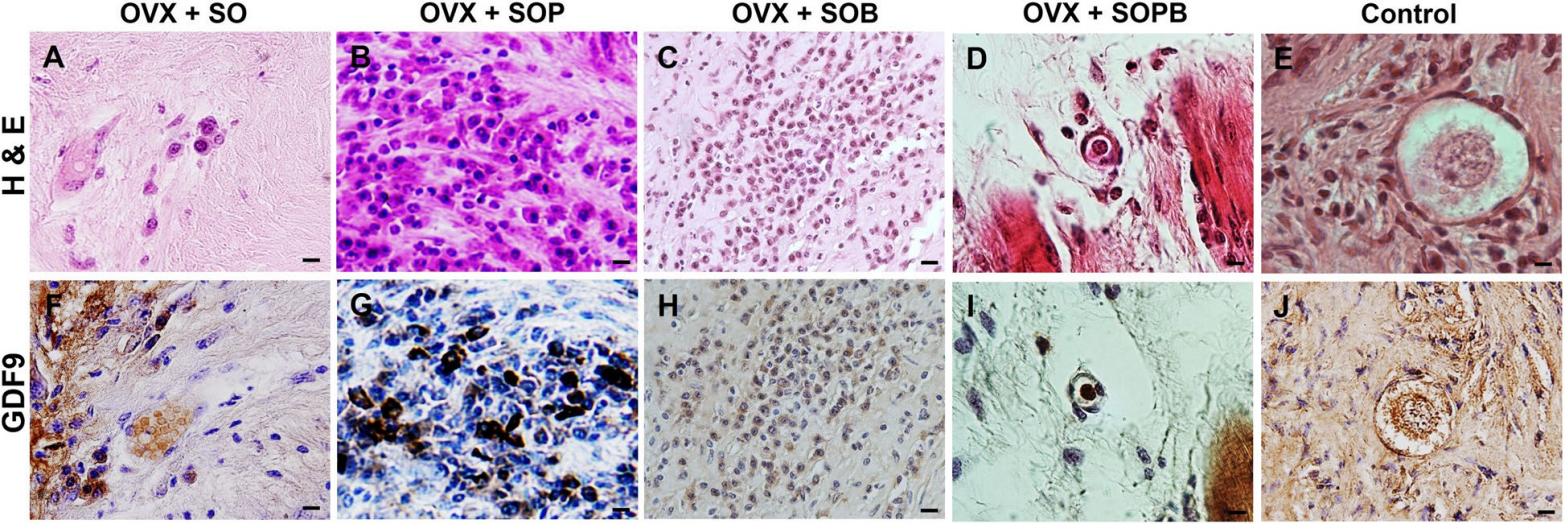
H & E and Immunohistochemical staining for antibodies against Growth differentiation factor 9 (GDF9*)* protein in artificial ovaries (Scale bars: 10 µm)

**Fig. 14:**
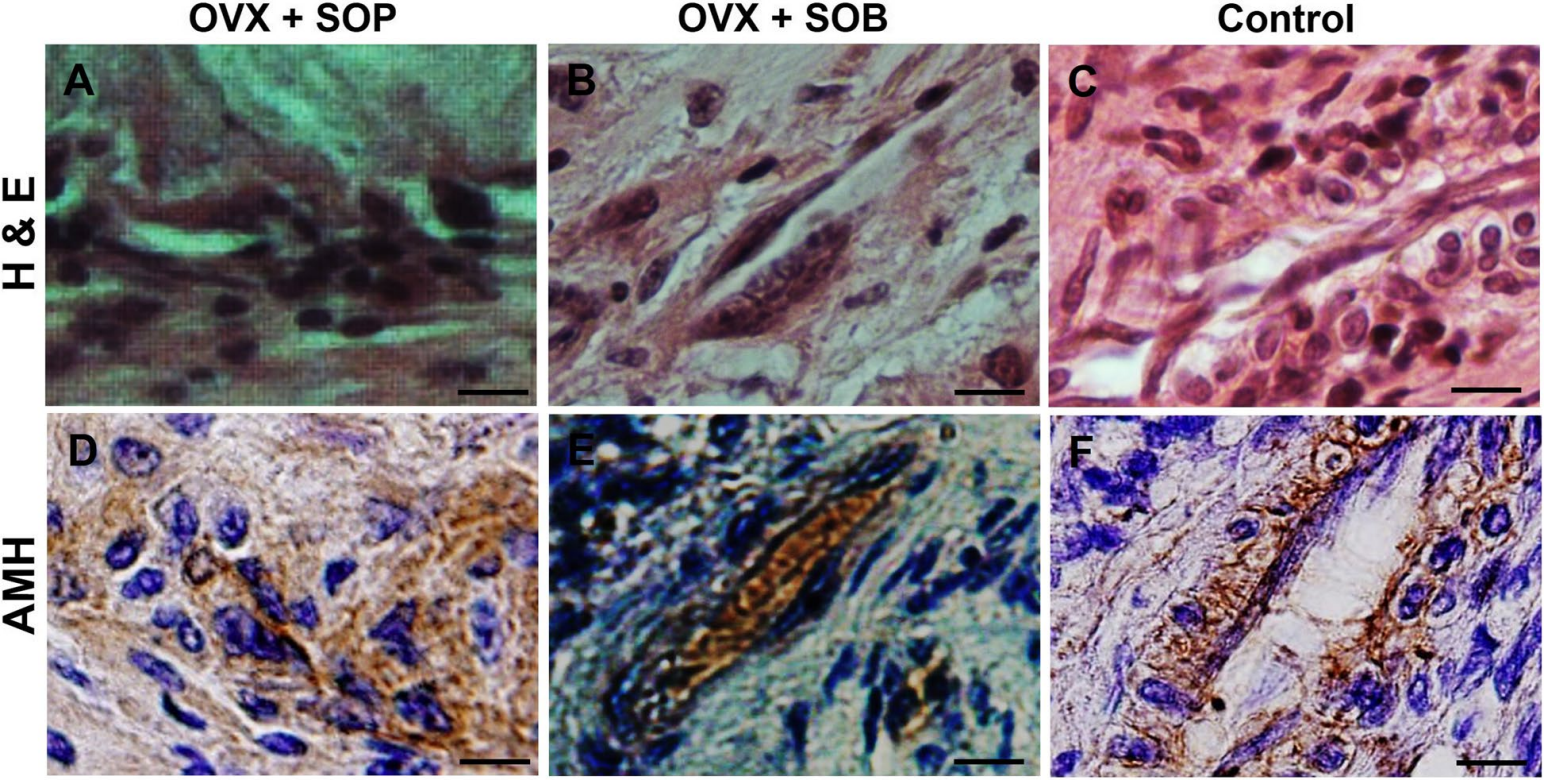
H & E and Immunohistochemical staining for antibodies against Anti-müllerian hormone (AMH) protein in artificial ovaries (Scale bars: 10 µm)

To verify previous results, the expressions of specific proteins associated with oocyte (ZP3 and GDF9) and granulosa (AMH) cells were investigated using immunohistochemistry assay in the sections of artificial ovaries in all groups. Also, the expression of the GFP protein, PCNA cell proliferation protein and VEGF were evaluated. As shown in Figs. 9, 10, 11, 12, 13, 14, all proteins were expressed more or less in all groups, but Amh protein was expressed only in the SOP and SOB groups.

### Quantitative comparison of the proteins

**Fig. 15:**
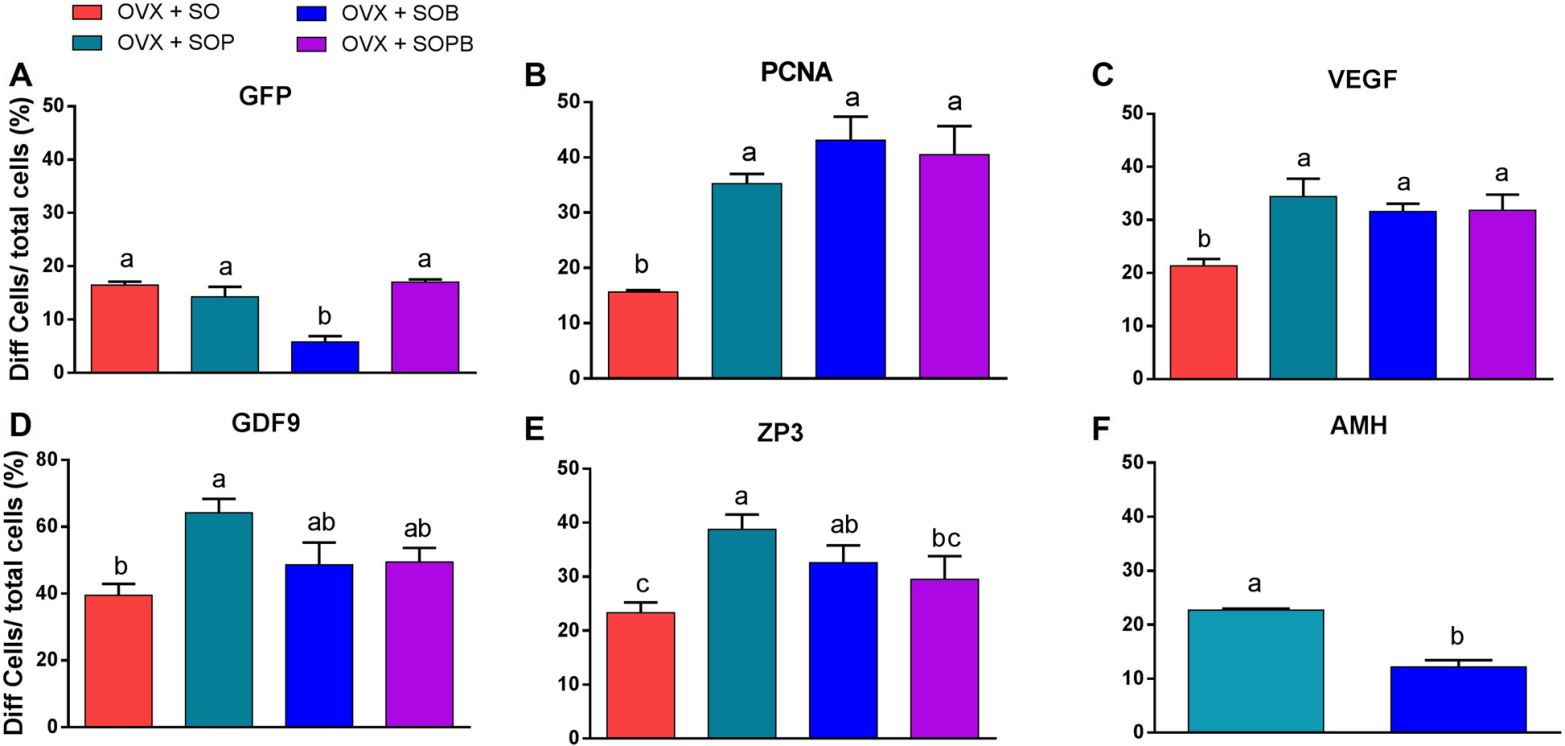
The graphs show the quantitatively analyses from immunohistochemistry staining results. Values are given as mean ± SEM. (a-b) Groups followed by the same letter are not significantly different at *p* < 0.05

To statistically compare protein expression between experimental groups, the qualitative results of immunohistochemical staining for each protein were counted using the Image J software and are written as percentages in Fig. 15. As shown in the figure, the expression of the anti GFP protein was observed in all groups, but SO, SOP and SOPB groups showed significant increases compared to the SOB group (Fig. 15A). Protein expressions related to cellular proliferation and angiogenesis were observed in all groups, but SOP, SOB and SOPB groups showed higher values compared to the SO group (P < 0.05) (Fig. 15B, C). A significant increase in the expression of Gdf9 (Zp3) in SOP samples was observed compared to the SO (SO and SOPB) groups (Fig. 15D, E). On the other hand, expression of Amh protein was seen in only SOP and SOB, presenting significant difference between the two groups (*P* < 0.05) (Fig. 15F).

### Macroscopic and microscopic alterations in morphological features of transplanted constructs

**Fig. 16:**
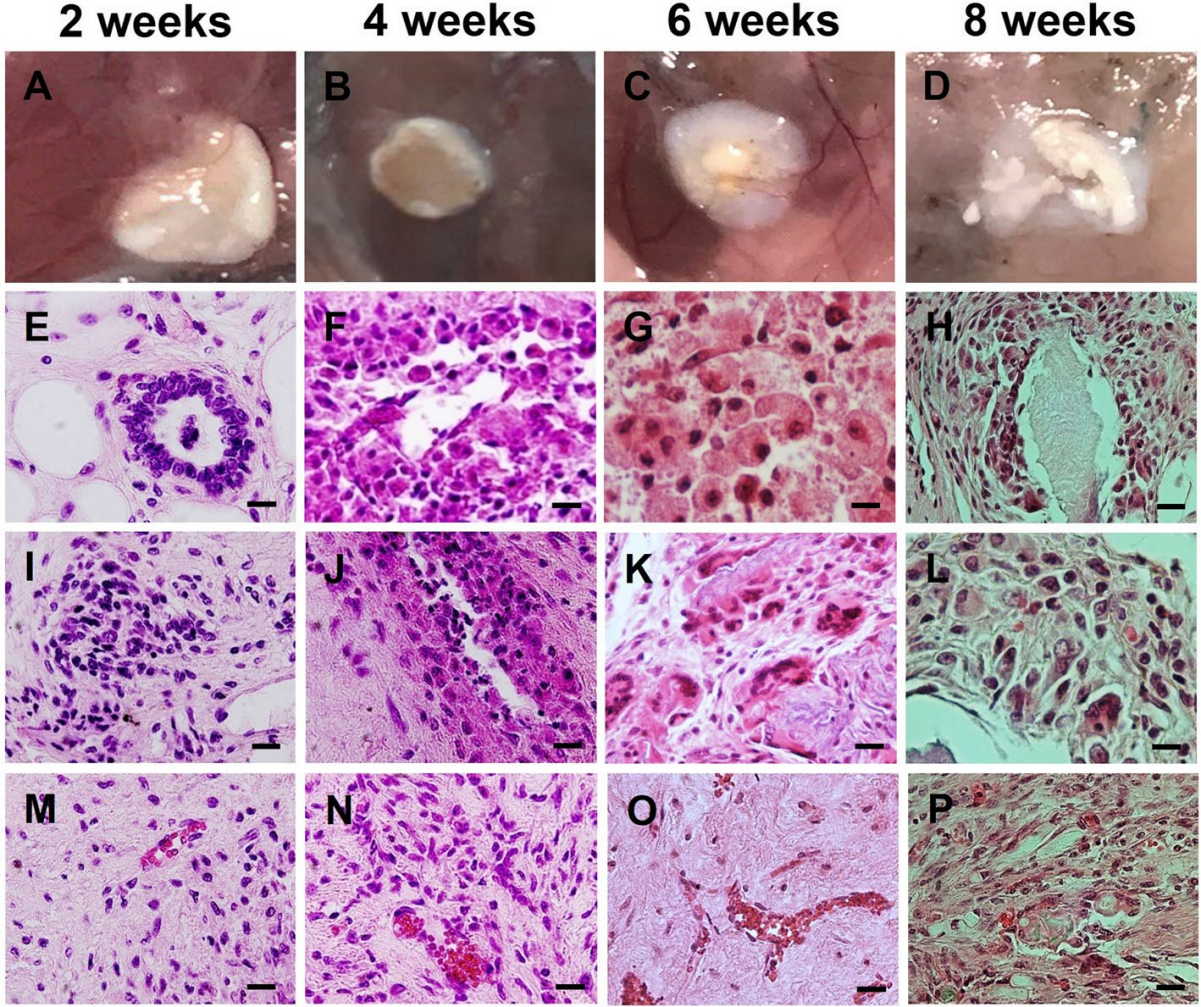
(**A**-**D**) Macroscopic appearance and (**E**-**P**) H & E staining of recellularized human ovarian scaffold with OSCs + PMSCs for 2 (**A**, **E**, **I**, **M**), 4 (**B**, **F**, **J**, **N**), 6 (**C**, **G**, **K**, **O**) and 8 (**D**, **H**, **L**, **P**) weeks after transplantation to Nude mice (Scale bars: 20 µm)

In order to compare the mice with immunosuppressed and active immune systems, the ovarian scaffold was seeded with OSCs + PMSCs and transplanted to sub-peritoneal region of the immunosuppressed mice for 2, 4, 6 and 8 weeks. To check the graft’s health, the gross histology of each graft was studied in the mentioned intervals. Angiogenesis was observed in the second week of transplantation and continued to rise up to 8^th^ week. Angiogenesis in the transplantation site was evident at the sixth week and clearly seen in most artificial ovaries after eight weeks of transplantation into NMRI mice. The formation of fibrous capsule was seen in four weeks. The graft size did not change in 2 and 4 weeks, but it was a little smaller at 6 weeks, and this reduction was more pronounced during the 8^th^ week (Fig. 16A-D). The appearance of artificial ovary fibrosis and shrinkage in NMRI mice was very low; in one of the transplanted artificial ovary, apparently half the graft had remained undetected on the sixth week. On the other hand, H & E staining showed that follicle-like structures and cellular assemblages were presented in both immunosuppressive mice and NMRI mice after 2 weeks (Fig. 16E-P). At 6 weeks old artificial ovaries, round cells were detected that were similar to the oocytes (Fig. 16G).

## Discussion

There are several methods of fertility preservation in young women and girls who suffer from gonadotrophic cancer treatment; some such preservation methods are embryo or egg freezing, ovarian transplantation and IVM. Each method has its own advantages and disadvantages, and may not be suitable for all cancer patients. According to the new guidelines, ovarian tissue cryopreservation is one of the strategies for fertility preservation in women affected by cancer [20]. Although ovarian transplantation has been successful, the possibility of cancer cells returning from tissue stroma to transplantation sites cannot be overlooked. Given the increasing number of young girls surviving cancer, there is a need for fertility preservation procedures that can avoid the recurrence of malignancy currently seen in cryopreserved ovarian transplantation.

In recent years, many research groups have been developing artificial systems for ovarian tissue with the aim of restoring fertility in women who may not be amenable to ovarian transplantation due to risks of cancer recurrence or suffering from the aftereffects of surgery. Ovarian tissue engineering by an ovarian decellularized scaffold was initially used by Laronda et al. to restore fertility in ovariectomized mice [21].

In the current study, we have shown new and practical models and a dynamic method for stem cell seeding. The scaffold was obtained from the ovarian decellularized tissue of trans-sexual people who were neither sick nor under chemotherapy or radiotherapy. In the present study, a dynamic method for PMSCs seeding into 5 × 5 × 1 mm^3^ pieces of ovary was developed. To compare the differentiation potential of PMSCs and BMSCs for oogenesis initiation and OSCs induction for folliculogenesis, the human ovarian decellularized scaffolds were categorized into four types: SO, SOP, SOB and the SOPB were seeded with labeled stem cells for one week in ex vivo culture and formed the engineering constructs. Each of the constructs was transplanted to the sub-peritoneum of ovariectomized mice. The onset of oogenesis and puberty was evaluated and compared in different groups after 8 weeks. Before and after the seeding process, it was seen that OSCs (green), PMSCs (red) and BMSCs (purple) were hosted into the scaffold, and observed after the duration of transplantation in the artificial ovary. Zeng et al., reported that the transplantation of primordial germ cells into old mice causes the onset of oogenesis [22]. It was believed that increasing the length of the transplantation period (more than 4 weeks), would cause follicular apoptosis. Kidney capsule and ovarian bursa have shown similar results. In the present research, in all groups in general, and in the SOP and SOB groups in particular, follicle-like structures are formed that often have one or more cells in the center. Unlike earlier studies that reported that ovarian mammals have a steady number of primordial follicles produced during the embryonic period and without adult germ stem cells that are responsible for follicle recovery, it is now believed that the ovary is a dynamic organ, very similar to the testis in terms of germ cells production, and continues to produce germ cells until puberty [23]. It is likely that oogonial stem cells residing in the ovary are differentiated into germ cells and enter the follicular development stage. Therefore, oogonial stem cells play an important role in the survival and function of the ovary and fertility. Analysis of the cell lineage in the ovarian rodent has suggested that oogenesis probably continues after the fetal period [9]. It is claimed that the sources of germ stem cells were non-ovarian, but this may indicate the nature of stem cells from bone marrow and blood [10].

MSCs are also seen to help oogenesis in our study. White et al., believed that a group of OSCs originates from the ovarian surface epithelium [24]. These OSCs express germ cell markers (Ddx4). In present study, MSCs differentiate into ovarian cell-like cells and by producing growth factors and cytokines, induce OSCs to produce oocyte-like cells and oogenesis. OSCs need to interact with somatic cells to differentiate into oocytes. Ovarian somatic cells play a significant role in germ cells development and may play a role in the survival and differentiation of OSCs [14]. Mouse ESCs expressed oocyte specific genes when co-cultured with granulosa cells, but in granulosa conditioned medium, these genes were not expressed [25]. These results confirm the role of somatic cells in oogenesis, although cell–cell interactions between somatic cells and stem cells may be necessary for oogenesis.

From this study, it is seen that MSC may also play the role of ovarian somatic cells for OSCs. PMSCs are isolated from peritoneum mesothelium. Ovarian surface epithelium (OSE) also originates from peritoneum mesothelium during mesenchymal to epithelial transformation and its function is cell regeneration and proliferation after ovulation during the reproductive period [26]. Parrot et al., showed that human and cow OSE cells express c-kit receptors, kit-ligand and stem cell factor (SCF) in vitro [27]. In line with our observations, Bukovsky et al., showed that OSE can be a bipotent source for producing both granulosa and germ cells [28]. Therefore, our hypothesis is that PMSCs have the ability to differentiate into ovarian cell-like cells similar to OSE cells. In an earlier study, PMSCs were found to differentiate into oocyte, germ and granulosa cell-like cells when they were exposed to human follicular fluid and cumulus cells conditioned media in vitro and in adherent culture medium [16]. On the other hand, it was also observed that PMSCs exposed to the ovarian niche and ECM signaling differentiated into primordial germ cell-like cells and lost their mesenchymal features after one week of culture. The ECM compounds and ultrastructure regulate the achievement of cytokines and nutrients, as well as induce mechanotransduction signals that are necessary for tissue formation. For example, human endothelial cells attach to pig small intestine sub mucosa better than ECM-coated plastic dishes [29].

Hoganson et al., showed that pig mesothelium scaffold in addition to protecting ECM proteins, including collagen 4, fibronectin and laminin, significantly preserved growth factors including VEGF, bFGF, and TGF-β that may be involved in post-transplantation tissue regeneration [30]. The secretion of VEGF and bFGF leads to angiogenesis and TGF-β results in collagen production in the scaffold [31]. Hoganson and colleagues also showed that Vegf increases significantly after using of mesothelium scaffold conditioned medium co-cultured on human fibroblasts. It was believed that in scaffold conditioned medium there are unknown solution factors that stimulate fibroblasts to Vegf secretion. It is also believed that the capability of the scaffold to stimulate cytokine production from other cells such as MSCs can be studied. Human adrenocortical cells seeded into pig adrenal decellularized scaffold have shown better proliferation after close contact with the ECM, and produced more cortisol [32]. In some approaches, liver, lung, kidney, and heart of the rodents were also decellularized and recellularized to restore measurable amount of function, including metabolic activity and oxygen exchange after transplantation [33–36].

The current study offers a new approach to restore ovarian endocrine function using regenerative medicine in human ovarian scaffold. It is seen that the cells survive after transplantation and show high proliferation capacity, and this could be due to the presence of blood vessels in the transplanted scaffold. The proliferation capability has a direct relationship with vascularization. Results of immunohistochemistry staining on cell proliferation proteins (PCNA) and vascularization (VEGF) indicated significant increase in the SOP, SOB and SOPB groups compared to SO group. Therefore, it is likely that the groups with high cell proliferation have an increase in blood perfusion to the scaffold and vice versa. The blood vessels around scaffold are clearly visible in Fig. 11 A-D especially in the SOB group. It is also seen that gene expression related to angiogenesis (Vegf, CD34), in SOB group, Vegf increased significantly compared to SO and SOP groups and CD34 gene expression increased analytically compared to just SOP group. On the other hand, in SO and SOP groups, CD34 expression is seen to be significantly increased compared to SOP. Based on this idea that BMSCs are involve in angiogenesis, it is likely that BMSCs, along with OSCs, increase angiogenesis within the scaffold.

Edessy et al., observed that ovarian function improved both in hormone and follicular development after autologous BMSCs injection into rat peritoneal cavity with POF-induced cyclophosphamide [37]. In the current research, stem cells is seen to increase serum estradiol levels in all groups and the rise in estradiol is significant in the SOPB group in comparison to the other groups. Gene expression studies show that the expression level of theca cell marker (Lhr) in the SOPB group is significantly higher than the other groups. Thus, it can be said that theca cells in this group have more steroidogenic function. It is possible that in other samples, because of the low expression of Lhr or its ineffectiveness, the quantity of estradiol is low.

Amh is a TGF-β family and is often expressed in granulosa cells more than in oocytes and ovarian stromal cells. It is supposed that Amh has the highest expression in granulosa cells of the preantral and small antral follicles [38]. In the present study, Amh are produced in all groups and this observation is not significantly different among the groups. As with gene expression, there is no significant difference in Amh expression between any of groups. Immunohistochemistry results indicate that Amh protein is only expressed in SOP and SOB groups and the expression in SOP is significantly higher than in SOB. Folliculogenesis is related to Amh expression in granulosa cells. Amh regulates follicular recruitment by limiting the activated follicles. Our study shows that in H & E staining, follicle-like structures are often formed in SOP and SOB groups. Immunohistochemistry data also confirm this claim, special in groups with increasing AMH protein production.

The secondary sexual characteristics (vaginal opening) were monitored in recipient groups, OVX and natural cycle in control mice (animals with no transplantation). Vaginal opening represents oogenesis induction and increasing estradiol production from ovary induces this characteristic in mice. The vaginal orifice is seen to be well opened in SOP, SOB and SOPB groups, but in SO group, in all cases except one, the vagina remained close at a similar time. A direct correlation is also seen between increased Lhr gene expression, serum estradiol hormone and vaginal opening in the SOPB group.

Liu et al. implanted mouse ovarian tissue fragments into pig ovarian decellularized tissue and cultured them ex vivo for 9 days and then evaluated recellularization potential [39]. Interestingly, granulosa cells not only successfully penetrated into the scaffold but also improved endocrine function. The migration of cultured granulosa cells into scaffold in vitro has also been reported [39]. In the present study, decellularized scaffold and ovarian niche are seen to support the growth of oocyte and germ cell-like cells. In examining the gene expression of germ cell-like cells (Ddx4 +), in SOP group, the increase is higher than in other groups but not significantly so. In fact, PMSCs and OSCs in this group may be differentiated into germ cell-like cells; hence, the expression of Ddx4 was higher than the other groups. On the other hand, the expression of Gdf9 and Zp3 genes do not differ significantly among the groups.

In protein expression levels, Gdf9 and Zp3 markers are significantly increased in the SOP group compared to SO (SO and SOPB). Indeed, PMSCs along with OSCs, are involved in the production of oocyte-like cells and oogenesis induction in transplanted ovarian scaffold. Immunohistochemistry results of the current study suggest that oogenesis signs are more evident in the experimental groups with MSCs and OSCs. Thus, we can say that MSCs secrete factors that trigger the onset of differentiation and growth of OSCs. It is believed that they also stimulate angiogenesis. However, MSCs may also differentiate into granulosa, theca and or oocyte cells along this pathway.

Johnson et al. believed that bone marrow and peripheral blood stem cells renewed ovarian follicles and returned fertility in chemically treated mice ovaries [10]. By examining the expression of Ddx4, Johnson et al. showed that bone marrow stem cells have the ability to differentiate into germ cells [10]. Soares et al. suggested that ovarian stromal cells or ovarian tissues secrete factors that are involved in the initial activation, growth, and angiogenesis of primordial follicles. Therefore, a natural microenvironment is required for initial activation [64].

In the present study, transplant recipients were not immunosuppressed and the interaction of immune cells, including macrophages and lymphocytes, will be investigated in the future. Researchers believe that host immune responses can affect the biocompatibility of xenogeneic decellularized tissues. Liu et al. showed that the surface markers of the immune system (CD68, CD86 and CD3) have significant penetration in cellularized tissues in comparison to decellularized tissues after subcutaneous implantation. Liu et al., believe that the response of M1 macrophage is inhibited in cellularized tissues as against decellularized scaffolds and T cell proliferation is also reduced. Not a single case of rejection was observed in the H & E staining. Of course, in some tissues, there were cells the identity of which were unknown to us. Therefore, in addition to transplantation to NMRI mice, one group was transplanted into immunosuppressed mice. After examining the gross and H & E, there were no significant differences between them and the NMRI mice and follicle-like structures were seen from the second week onwards.

Fishman et al. suggested that decellularized scaffold have anti-inflammatory and immunosuppressive effects [40]. These effects may explain why ovarian scaffold in the Liu study did not show the penetration of M1 macrophages and CD3^+^ T lymphocytes after 4 weeks of transplantation. Its mechanism may be due, in part, to the degradation of MHCI, II molecules after decellularization [41].

The future of a safe artificial ovary for human use relies on regenerative medical techniques. Stem cells that have been seeded into ovarian decellularized scaffold of sick or healthy people can be important for this purpose. In fact, it is known that stem cells differentiate into tissue-specific cells. It was shown in this study that MSCs and OSCs in ovarian scaffold have the ability to differentiate into ovarian cell lines and induce oogenesis. The clinical outcome of our study is that the transmission of residual cancer cells can be avoided by using ovarian cells derived from stem cells, along with ovarian decellularized scaffold from an allogeneic source such as human trans-sexual or cadaver.

## Conclusions

An artificial ovarian tissue can potentially restore fertility in women who may not be able to have ovarian transplantation because of the risk of recurrent cancer. In the current study, we have shown a new approach of stem cells seeding in a scaffold obtained from ovarian decellularized tissue. Follicle-like structures are seen to be formed as one or more cells in the artificial ovarian tissue center. It is likely that oogonial stem cells residing in the ovary are differentiated into germ cells and enter the follicular development stage. Thus, oogonial stem cells can play an important role in the survival and function of the ovary and ultimately in fertility preservation.
